# Supplementary figures and images for: Genetic interaction mapping reveals functional relationships between peptidoglycan endopeptidases and carboxypeptidases
Source: PLoS Genet. 2024 Apr 10;20(4):e1011234. doi: 10.1371/journal.pgen.1011234 (PMC11034669; doi:10.1371/journal.pgen.1011234)

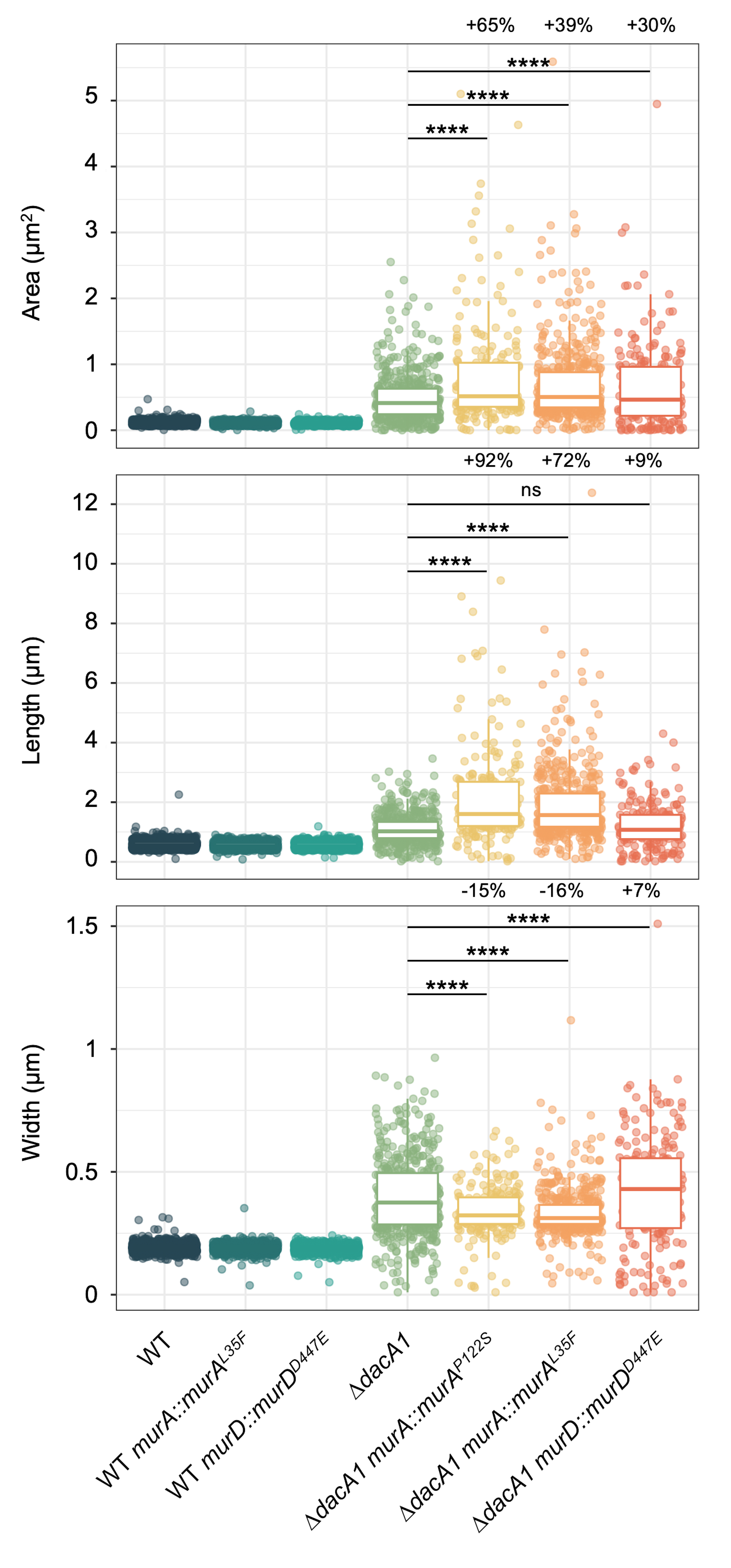

Supplement: S6 Fig — Cells were grown as described in Fig 5B. Images were segmented using Omnipose and then analyzed for their area, length, and width using MicrobeJ. Statistical significance was determined using Welch’s t-test. The significance level was denoted as ns, not significant; *, P < 0.05; **, P < 0.01; ***, P < 0.001; ****, P < 0.0001. (TIF) [file pgen.1011234.s006.tif]

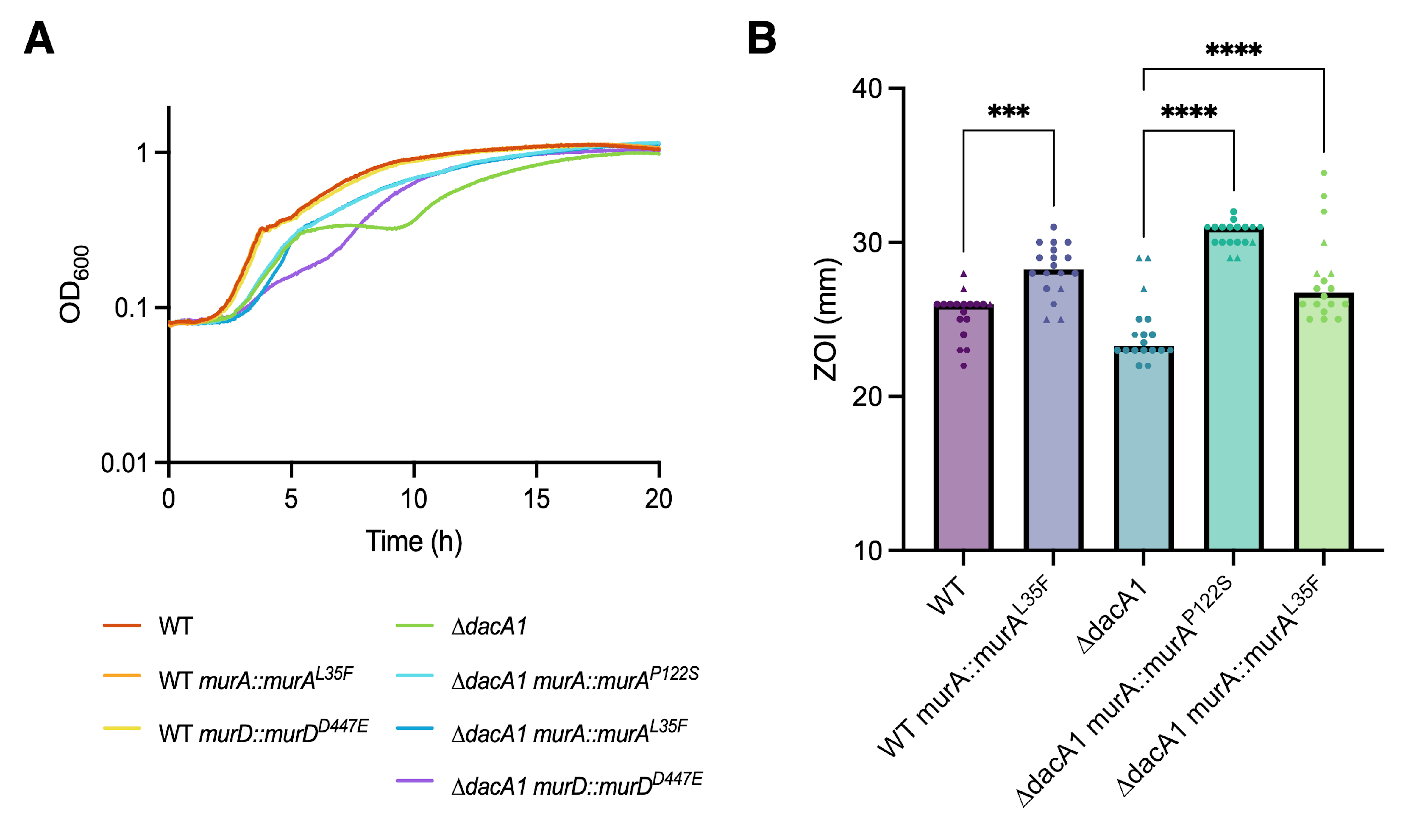

Supplement: S7 Fig — (A) Overnight cultures were diluted 1:10,000 and grown on LB broth for 20 hours, OD600 measurements were taken every 2 minutes. (B) Fosfomycin sensitivity was measured in the different murA mutants. Cultures of the indicated strains were plated evenly (∼108 colony forming units [cfu]) on LB agar. A filter disk containing 10 μL of fosfomycin (50mg/mL) was placed in the center of the plate followed by incubation at 30° C for 24h. Bars indicate zone of inhibition (ZOI) in mm. Statistical significance was calculated with a one-way ANOVA. Significance level was denoted as ns, not significant; *, P < 0.05; **, P < 0.01; ***, P < 0.001; ****, P < 0.0001. (TIF) [file pgen.1011234.s007.tif]

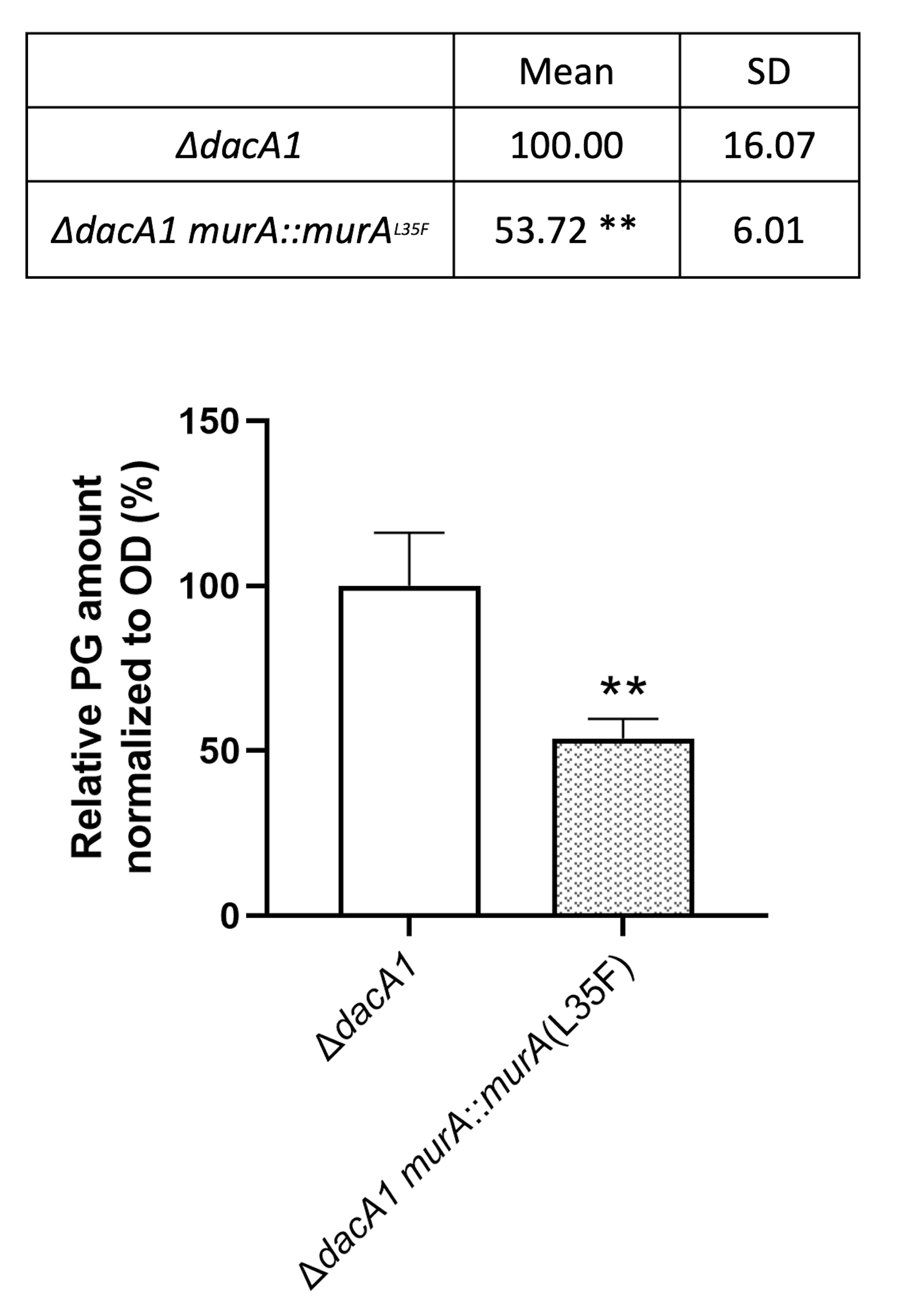

Supplement: S8 Fig — Relative PG amount was calculated by measuring the total area of the chromatogram and normalized to the OD at the time of harvesting. The relative percent was calculated by dividing the normalized total area by the average of the control sample and multiplying it by 100. Statistical significance was calculated using an unpaired t-test analysis (*P-value < 0.05, **P-value < 0.01, ***P-value < 0.001). (TIF) [file pgen.1011234.s008.tif]

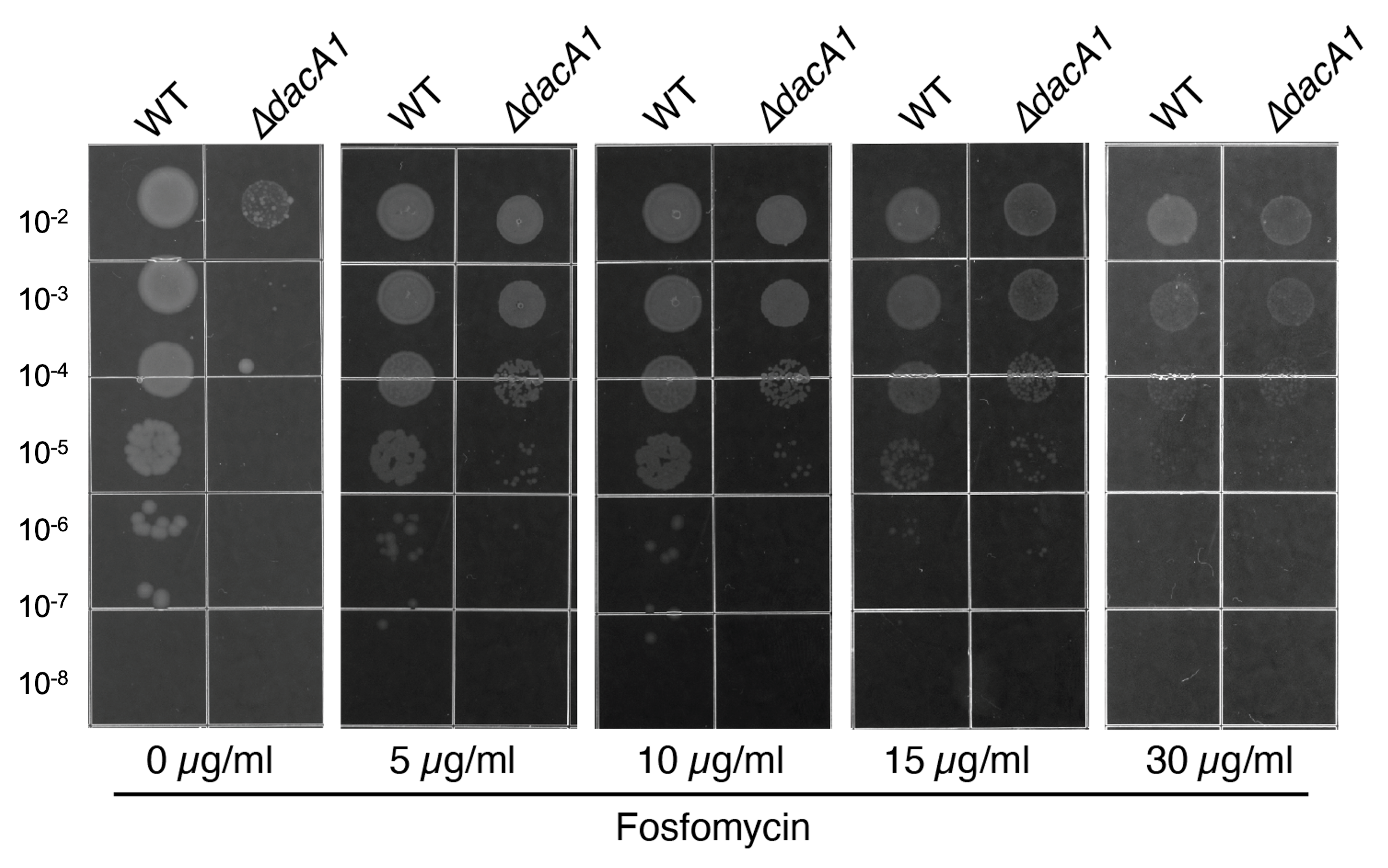

Supplement: S9 Fig — Overnight cultures were plated on LB plates with increasing concentrations of fosfomycin (5μg/mL, 10μg/mL, 15μg/mL, 30μg/mL) and incubated overnight at 30°C. (TIF) [file pgen.1011234.s009.tif]

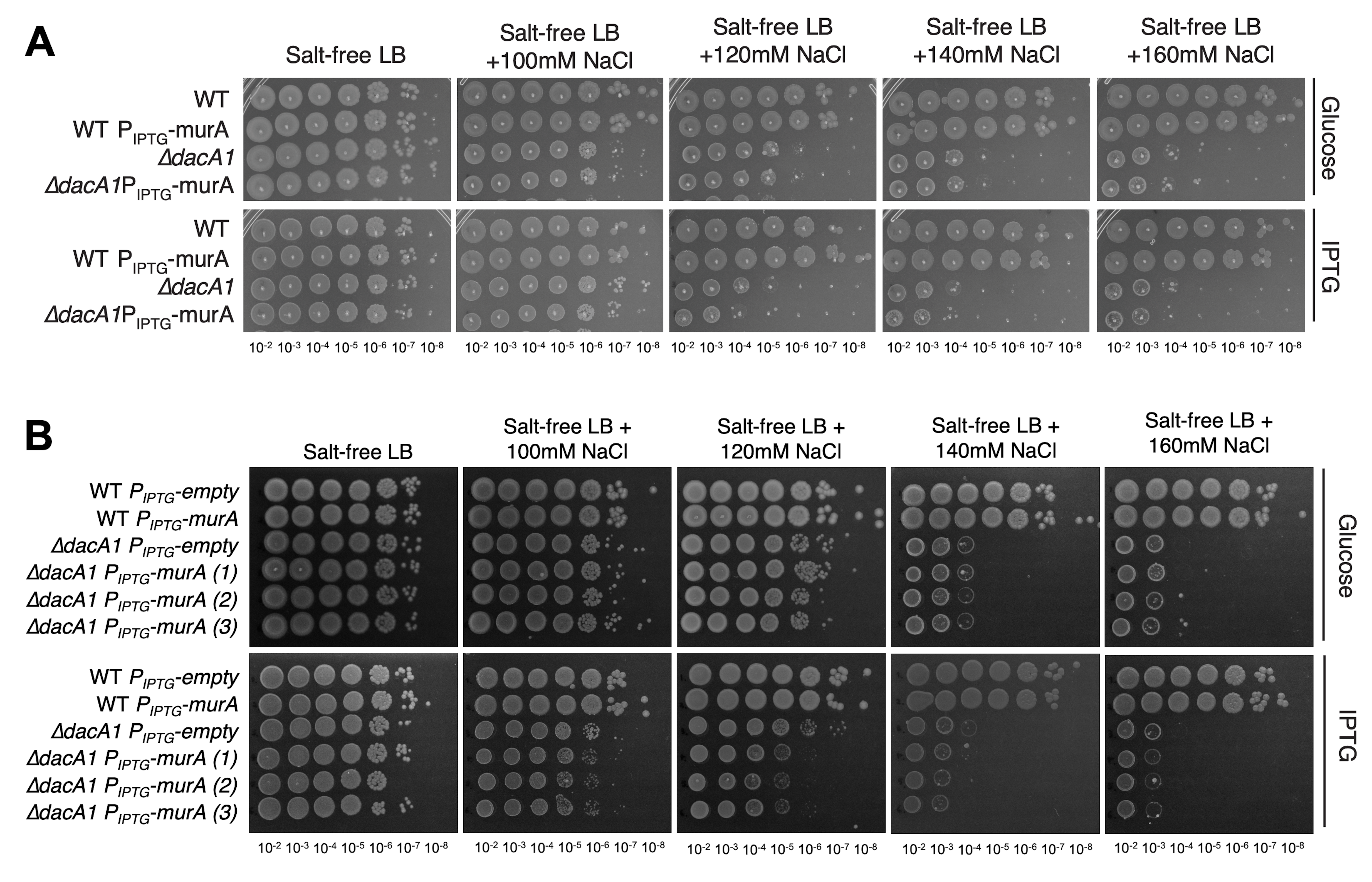

Supplement: S13 Fig — Overnight cultures of WT, ΔdacA1 and both backgrounds harbouring an IPTG inducible copy of murA were plated on salt-free LB with increasing concentrations of NaCl (120mM, 140mM and 160mM) and either 0.2% glucose or 1mM IPTG, and incubated at 30°C overnight. (A) Replicate 2 (B) Replicate 3 with empty pTD101 vector integration as negative control and 3 different clones of ΔdacA1 PIPTG-murA. (TIF) [file pgen.1011234.s013.tif]
