# Supplementary material for: Genetic interaction mapping reveals functional relationships between peptidoglycan endopeptidases and carboxypeptidases
Source: PLoS Genet. 2024 Apr 10;20(4):e1011234. doi: 10.1371/journal.pgen.1011234 (PMC11034669; doi:10.1371/journal.pgen.1011234)
Supplement: S2 Table — (DOC) [file pgen.1011234.s016.doc]

**Supplemental Table 2. Suppressor screen mutation summary (*ΔdacA1* on LB)**

B: stands for big colonies isolated from the screen

S: stands for small colonies isolated from the screen

**Big Colonies (B) Replicates 1, 2 and 3.**

| **Suppressor** | **Mutation** | **Gene** | **Annotation** |
| --- | --- | --- | --- |
| **1.B1** | Δ106 bp  coding (631‑736/1794 nt) | *VC_0393* ← | O-antigen_ligase-related |
|  | Δ3 bp  coding (535‑537/648 nt) | VC_A0170 ← | Aerotolerance-related protein BatD |
|  | Δ1 bp  coding (533/648 nt) | VC_A0170 ← |  |
|  | G→T  P177P (CCC→CCA) | VC_A0170 ← |  |
| **1.B2** | Δ1,261 bp | *[VC_0255]*–*[VC_0258]* | VC_0255: [O-antigen methyltransferase WbeT](https://www.pnas.org/content/pnas/118/7/e2018032118.full.pdf)  VC_0256: Transposase OrfAB, subunit A  VC_0257: Transposase OrfAB, subunit B  VC_0258: [WbeT](https://www.sciencedirect.com/science/article/pii/S0925443904001000) |
|  | Δ6,344 bp | *[VC_0391]–[VC_0394]* | VC_0391: Aspartokinase  VC_0392: Aminotransferase, class V  VC_0393: O-antigen_ligase-related  VC_0394: UvrABC system protein A |
| **1.B4** | Δ106 bp  coding (631‑736/1794 nt) | *VC_0393* ← | O-antigen_ligase-related |
|  | Δ3 bp  coding (535‑537/648 nt) | VC_A0170 ← | Aerotolerance-related protein BatD |
|  | Δ1 bp  coding (533/648 nt) | VC_A0170 ← |  |
|  | G→T  P177P (CCC→CCA) | VC_A0170 ← |  |
| **1.B5** | Δ106 bp  coding (631‑736/1794 nt) | *VC_0393* ← | O-antigen_ligase-related |
| **1.B6** | Δ106 bp  coding (631‑736/1794 nt) | *VC_0393* ← | O-antigen_ligase-related |
| **1.B7** | Δ106 bp  coding (631‑736/1794 nt) | *VC_0393* ← | O-antigen_ligase-related |
| **1.B8** | Δ106 bp  coding (631‑736/1794 nt) | *VC_0393* ← | O-antigen_ligase-related |
| **2.B1/B4** | Δ1,261 bp | *[VC_0255]*–*[VC_0258]* | VC_0255: [O-antigen methyltransferase WbeT](https://www.pnas.org/content/pnas/118/7/e2018032118.full.pdf)  VC_0256: Transposase OrfAB, subunit A  VC_0257: Transposase OrfAB, subunit B  VC_0258: [WbeT](https://www.sciencedirect.com/science/article/pii/S0925443904001000) |
|  | Δ106 bp  coding (631‑736/1794 nt) | *VC_0393* ← | O-antigen_ligase-related |
| **2.B2** | Δ1,261 bp | *[VC_0255]*–*[VC_0258]* | VC_0255: [O-antigen methyltransferase WbeT](https://www.pnas.org/content/pnas/118/7/e2018032118.full.pdf)  VC_0256: Transposase OrfAB, subunit A  VC_0257: Transposase OrfAB, subunit B  VC_0258: [WbeT](https://www.sciencedirect.com/science/article/pii/S0925443904001000) |
|  | Δ106 bp  coding (631‑736/1794 nt) | *VC_0393* ← | O-antigen_ligase-related |
| **2.B3** | Δ106 bp  coding (631‑736/1794 nt) | *VC_0393* ← | O-antigen_ligase-related |
| **2.B5** | Δ106 bp  coding (631‑736/1794 nt) | *VC_0393* ← | O-antigen_ligase-related |
| **2.B6** | Δ6,344 bp | *[VC_0391]–[VC_0394]* | VC_0391: Aspartokinase  VC_0392: Aminotransferase, class V  VC_0393: O-antigen_ligase-related  VC_0394: UvrABC system protein A |
| **2.B7** | Δ106 bp  coding (631‑736/1794 nt) | *VC_0393* ← | O-antigen_ligase-related |
| **2.B8** | Δ106 bp  coding (631‑736/1794 nt) | *VC_0393* ← | O-antigen_ligase-related |
|  | C→A  E64* (GAA→TAA) | VC_2630 ← | pilQ: Fimbrial assembly protein |
| **3.B1** | Δ106 bp  coding (631‑736/1794 nt) | *VC_0393* ← | O-antigen_ligase-related |
| **3.B2** | Δ6,344 bp | *[VC_0391]–[VC_0394]* | VC_0391: Aspartokinase  VC_0392: Aminotransferase, class V  VC_0393: O-antigen_ligase-related  VC_0394: UvrABC system protein A |
| **3. B3** | Δ1,261 bp | *[VC_0255]*–*[VC_0258]* | VC_0255: [O-antigen methyltransferase WbeT](https://www.pnas.org/content/pnas/118/7/e2018032118.full.pdf)  VC_0256: Transposase OrfAB, subunit A  VC_0257: Transposase OrfAB, subunit B  VC_0258: [WbeT](https://www.sciencedirect.com/science/article/pii/S0925443904001000) |
|  | Δ106 bp  coding (631‑736/1794 nt) | *VC_0393* ← | O-antigen_ligase-related |
|  | (TGATAAAAAATTAG)1→2  intergenic (‑49/+60) | *VC_1840 ← / ← VC_1841* | VC_1840: 4HBT domain-containing protein  VC_1841: Hypothetical protein  *The mutation is right before the TolQRAB |
| **3. B4** | Δ106 bp  coding (631‑736/1794 nt) | *VC_0393* ← | O-antigen_ligase-related |
| **3.B5** | Δ106 bp  coding (631‑736/1794 nt) | *VC_0393* ← | O-antigen_ligase-related |
| **3.B6** | Δ106 bp  coding (631‑736/1794 nt) | *VC_0393* ← | O-antigen_ligase-related |
|  | Δ1 bp  coding (217/627 nt) | *VC_0523 ←* | csqA: quorum sensing gene  *aminotransferase |
| **3.B7** | Δ106 bp  coding (631‑736/1794 nt) | *VC_0393* ← | O-antigen_ligase-related |
| **3.B8** | Δ106 bp  coding (631‑736/1794 nt) | *VC_0393* ← | O-antigen_ligase-related |

**Small Colonies (S) Replicates 1, 2 and 3.**

| **Suppressor** | **Mutation** | **Gene** | **Annotation** |
| --- | --- | --- | --- |
| **1.S1** | A→C  D447E (GAT→GAG) | *VC_2403 ←* | MurD:UDP‑N‑acetylmuramoylalanine‑‑D‑glutamate ligase |
| **1.S2** | +C  coding (830/891 nt) | *VC_A0567 →* | VxrC |
| **1.S3** | C→T  A132T (GCT→ACT) | VC_2400 ← | MurC:UDP‑N‑acetylmuramate‑‑alanine ligase |
| **1.S4** | (T)7→6  intergenic (‑55/+1) | VC_0393 ← / ← VC_0394 | O-antigen_ligase-related/ excinuclease ABC, subunit A |
| **1.S6** | (T)7→6  intergenic (‑55/+1) | VC_0393 ← / ← VC_0394 | O-antigen_ligase-related/ excinuclease ABC, subunit A |
| **1.S7** | C→T  R79Q (CGA→CAA) | VC_0378 ← | Zur: zinc uptake regulation protein, putative |
| **1.S8** | C→T  R115W (CGG→TGG) | VC_A0079 → | ShyA |
| **2.S1** | C→A  Y471* (TAC→TAA) | *VC_2529 →* | RNA polymerase sigma‑54 factor |
| **2.S2** | - | *-* | - |
| **2.S3** | G→A  T32I (ACC→ATC) | *VC_1278 ←* | transcriptional regulator, MarR family |
| **2.S4** | Δ1 bp  coding (184/567 nt) | *VC_0238 ←* | WavK: O-acetyl transferase |
|  | Δ1,261 bp | *[VC_0255]–[VC_0258]* | VC_0255: [O-antigen methyltransferase WbeT](https://www.pnas.org/content/pnas/118/7/e2018032118.full.pdf)  VC_0256: Transposase OrfAB, subunit A  VC_0257: Transposase OrfAB, subunit B  VC_0258: [WbeT](https://www.sciencedirect.com/science/article/pii/S0925443904001000) |
| **2.S5** | (A)9→8  coding (613/1200 nt) | *VC_0269 →* | mannose‑6‑phosphate isomerase |
|  | (G)11→10  coding (232/738 nt) | *VC_1162 ←* | Zn_protease domain-containing protein |
| **2.S7** | T→C  L216P (CTG→CCG) | *VC_0477 →* | phosphoglycerate kinase |
| **2.S8** | Δ1,261 bp | *[VC_0255]*–*[VC_0258]* | VC_0255: [O-antigen methyltransferase WbeT](https://www.pnas.org/content/pnas/118/7/e2018032118.full.pdf)  VC_0256: Transposase OrfAB, subunit A  VC_0257: Transposase OrfAB, subunit B  VC_0258: [WbeT](https://www.sciencedirect.com/science/article/pii/S0925443904001000) |
|  | (TGATAAAAAATTAG)_1→2_  intergenic (‑49/+60) | *VC_1840 ← / ← VC_1841* | VC_1840: 4HBT domain-containing protein  VC_1841: Hypothetical protein  *The mutation is right before the TolQRAB |
|  | Δ1 bp  coding (1194/1464 nt) | *VC_2529 →* | RNA polymerase sigma‑54 factor |
| **3.S1** | G→A  T32I (ACC→ATC) | *VC_1278 ←* | Transcriptional regulator, MarR family |
| **3.S2** | Δ1 bp  coding (1318/1464 nt) | *VC_2529 →* | RNA polymerase sigma‑54 factor |
| **3.S3** | Δ1,261 bp | *[VC_0255]*–*[VC_0258]* | VC_0255: [O-antigen methyltransferase WbeT](https://www.pnas.org/content/pnas/118/7/e2018032118.full.pdf)  VC_0256: Transposase OrfAB, subunit A  VC_0257: Transposase OrfAB, subunit B  VC_0258: [WbeT](https://www.sciencedirect.com/science/article/pii/S0925443904001000) |
|  | Δ106 bp  coding (631‑736/1794 nt) | *VC_0393* ← | O-antigen_ligase-related |
|  | Δ21,125 bp | *VC_1452–VC_1478* | 25 genes |
| **3.S4** | G→A  R114* (CGA→TGA) | *VC_0866 ←* | transglycosylase, Slt family  mltF |
| **3.S5** | Δ106 bp  coding (631‑736/1794 nt) | *VC_0393* ← | O-antigen_ligase-related |
| **3.S6** | Δ6,344 bp | *[VC_0391]–[VC_0394]* | VC_0391: Aspartokinase  VC_0392: Aminotransferase, class V  VC_0393: O-antigen_ligase-related  VC_0394: UvrABC system protein A |
| **3.S7** | G→A  P122S (CCT→TCT) | *VC_2514 ←* | MurA:UDP‑N‑acetylglucosamine 1‑carboxyvinyltransferase |
| **3.S8** | C→A  L35F (TTG→TTT) | *VC_2514 ←* | MurA:UDP‑N‑acetylglucosamine 1‑carboxyvinyltransferase |
