# Supplementary material for: Genetic interaction mapping reveals functional relationships between peptidoglycan endopeptidases and carboxypeptidases
Source: PLoS Genet. 2024 Apr 10;20(4):e1011234. doi: 10.1371/journal.pgen.1011234 (PMC11034669; doi:10.1371/journal.pgen.1011234)
Supplement: S3 Table — (DOC) [file pgen.1011234.s017.doc]

**Supplemental Table 3. PG analysis**

| **Feature** | **Wild type** | ***ΔdacA1*** | ***ΔdacA1***  **lacZ::P_IPTG_-ShyA** | ***ΔdacA1***  **lacZ::P_IPTG_-ShyA^R115W^** | ***ΔdacA1***  **lacZ::P_IPTG_-ShyB** |
| --- | --- | --- | --- | --- | --- |
| **Crosslink** | 26.84 ±0.51 | 24.67 ±0.33 | 23.04 ±1.03** | 20.26 ±0.19*** | 20.64 ±0.59*** |
| **Pentapeptides** | 5.67 ±0.22 | 53.11 ±0.91 | 48.84 ±1.04*** | 46.46 ±1.29*** | 49.07 ±0.45*** |
| **M4** | 69.15 ±0.17 | 35.17 ±0.84 | 38.65 ±1.10*** | 40.60 ±1.03*** | 37.87 ±0.46*** |
| **M5** | 0.00 ±0.00 | 30.19 ±0.60 | 28.53 ±1.13*** | 29.38 ±1.01* | 31.89 ±0.51*** |
| **D44** | 17.04 ±0.46 | 5.88 ±0.24 | 5.97 ±0.32 | 5.66 ±0.19 | 5.68 ±0.19 |
| **D45** | 1.41 ±0.04 | 11.16 ±0.08 | 9.86 ±0.20*** | 7.56 ±0.18*** | 7.87 ±0.08*** |

Results of the comparison with the Δ*dacA1* (*P-value < 0.05, **P-value < 0.01, ***P-value < 0.001)
