# Supplementary material for: Genetic interaction mapping reveals functional relationships between peptidoglycan endopeptidases and carboxypeptidases
Source: PLoS Genet. 2024 Apr 10;20(4):e1011234. doi: 10.1371/journal.pgen.1011234 (PMC11034669; doi:10.1371/journal.pgen.1011234)
Supplement: S4 Table — (DOC) [file pgen.1011234.s018.doc]

***Supplemental Table 4. V. cholerae strains***

| **Strain #** | **Background** | **Description** | **Reference / Source** |
| --- | --- | --- | --- |
| MA1* | N16961 | Wild type El Tor strain | [1] |
| Common 87 | C6706 | Wild type El Tor strain | [2] |
| MA73 | N16961 | *lacZ*::P_IPTG_-*shyA^L109K^* | This study |
| MA259* | C6706 | *∆dacA1* | [3] |
| MA1078* | N16961 | *∆dacA1* | This study |
| MA282 | C6706 | *∆dacA1 lacZ::*P_IPTG_-*shyA^L109K^* | This study |
| MA670 | N16961 | *∆zur* | This study |
| MA730 | C6706 | *∆dacA1∆zur* | This study |
| MA1078 | C6706 | *∆dacA1∆zur lacZ::*P_IPTG_-*zur* | This study |
| MA788 | N16961 | *lacZ*::P_IPTG_-*shyB* | This study |
| MA810 | C6706 | *∆dacA1 lacZ*::P_IPTG_-*shyB* | This study |
| MA745 | N16961 | *lacZ*::P_IPTG_-*shyA* | This study |
| MA747 | N16961 | *lacZ*::P_IPTG_-*shyA^R115W^* | This study |
| MA749 | C6706 | *∆dacA1* *lacZ*::P_IPTG_-*shyA* | This study |
| MA751 | C6706 | *∆dacA1 lacZ*::P_IPTG_-*shyA^R115W^* | This study |
| MA1022* | N16961 | *murA::murA^L35F^* | This study |
| MA1026* | N16961 | *murD::murD^D447E^* | This study |
| MA1030* | C6706 | *∆dacA1 murA::murA^P122S^* | This study |
| MA1032* | C6706 | *∆dacA1 murA::murA^L35F^* | This study |
| MA1034* | C6706 | *∆dacA1 murD::murD^D447E^* | This study |
| MA940 | N16961 | *∆VC_A0040* | This study |
| MA971 | N16961 | *∆VC_A0040 ∆dacA1* | This study |
| MA1080 | N16961 | *∆VC_A0040 ∆dacA1 lacZ::*P_IPTG_-*vca0040* | This study |
| MA1207 | C6706 | *∆dacA1 lacZ::pTD101 empty* | This study |
| MA1209 | N16961 | *∆dacA1 lacZ::pTD101 empty* | This study |
| MA40 | N16961 | *lacZ::pTD101 empty* | This study |
| MA1305 | N16961 | lacZ:: Piptg-nlpC | This study |
| MA1307 | N16961 | lacZ:: Piptg-tagE1 | This study |
| MA1309 | N16961 | lacZ:: Piptg-tagE2 | This study |
| MA1311 | N16961 | lacZ:: Piptg-shyC | This study |
| MA1313 | N16961 | ∆dacA1 lacZ:: Piptg-nlpC | This study |
| MA1315 | N16961 | ∆dacA1 lacZ:: Piptg-tagE1 | This study |
| MA1317 | N16961 | ∆dacA1 lacZ:: Piptg-tagE2 | This study |
| MA1319 | N16961 | ∆dacA1 lacZ:: Piptg-shyC | This study |

*Strains were verified by whole genome sequencing.

References

1. Heidelberg, J. F. *et al.* DNA sequence of both chromosomes of the cholera pathogen Vibrio cholerae. *Nature* **406**, 477–483 (2000).
2. Thelin, K. H. & Taylor, R. K. Toxin-coregulated pilus, but not mannose-sensitive hemagglutinin, is required for colonization by Vibrio cholerae O1 El Tor biotype and O139 strains. *Infect. Immun.* **64**, 2853–2856 (1996).
3. Möll, A. *et al.* A D, D‐carboxypeptidase is required for Vibrio cholerae halotolerance. *Environ. Microbiol.* **17**, 527–540 (2015).
