# Supplementary material for: Genetic interaction mapping reveals functional relationships between peptidoglycan endopeptidases and carboxypeptidases
Source: PLoS Genet. 2024 Apr 10;20(4):e1011234. doi: 10.1371/journal.pgen.1011234 (PMC11034669; doi:10.1371/journal.pgen.1011234)
Supplement: S5 Table — (DOC) [file pgen.1011234.s019.doc]

**Supplemental Table 5. *E. coli* strains**

| **Strain #** | **Background** | **Description** | **Reference / Source** |
| --- | --- | --- | --- |
| MA57 | MFD λpir | pSC189 | [1] |
| Common 7 | DH5α λpir | Cloning strain | [2] |
| Common 8 | SM10 λpir | Conjugation strain | [3] |
| Common 10 | MFD λpir | Conjugation strain | [3] |
| MA547 | SM10 λpir | pTOX *dacA1* | This study |
| MA535 | SM10 λpir | pTOX *zur* | This study |
| MA38 | SM10 λpir | pTD101 empty | This study |
| MA261 | SM10 λpir | pTD101 *shyA^L109K^* | This study |
| MA1056 | SM10 λpir | pTD101 *zur* | This study |
| MA712 | SM10 λpir | pTD101 *shyB* | This study |
| MA678 | SM10 λpir | pTD101 *shyA* | This study |
| MA645 | SM10 λpir | pTD101 *shyA^R115W^* | This study |
| MA959 | MFD λpir | pTOX *murA^P122S^* | This study |
| MA962 | MFD λpir | pTOX *murA^L35F^* | This study |
| MA965 | MFD λpir | pTOX *murD^D447E^* | This study |
| MA1144 | SM10 λpir | pTD101 murA | This study |
| MA1060 | SM10 λpir | pTD101 VC_A0040 | This study |
| MA1283 | SM10 λpir | pTD101 nlpC | This study |
| MA1285 | SM10 λpir | pTD101 tagE1 | This study |
| MA1287 | SM10 λpir | pTD101 tagE2 | This study |
| MA1289 | SM10 λpir | pTD101 shyC | This study |
| MA875 | MFD λpir | pBADmob empty | This study |
| MA877 | MFD λpir | pBADmob mepM | This study |
| MA879 | MFD λpir | pBADmob mepM ∆dom1 | This study |
| MA1211 | MFD λpir | pBADmob shyB | This study |
| MA214 | MFD λ pir | pHLmob empty | This study |
| MA974 | MFD λ pir | pHLmob *shyA* | This study |
| MA976 | MFD λ pir | pHLmob *shyA^R115W^* | This study |
| MA1052 | BL21 (DE3) | pET28a murA | This study |
| MA1074 | BL21 (DE3) | pET28a murA^P122S^ | This study |
| MA1076 | BL21 (DE3) | pET28a murA^L35F^ | This study |
| MA1291 | BL21 (DE3) | pET28a murC | This study |
| MA1294 | BL21 (DE3) | pET28a murC^A132T^ | This study |
| CS109 | CS109 | Parental strain W1485 *glnV rpoS rph* | [4] |
| CS446-1 | CS109 | *∆dacA ∆dacB ∆dacC ∆pbpG* | [4] |
| MA984 | CS109 | pHLmob *empty* | This study |
| MA986 | CS109 | pHLmob *shyA* | This study |
| MA988 | CS109 | pHLmob *shyA^R115W^* | This study |
| MA994 | CS109 | CS446-1 pHLmob empty | This study |
| MA996 | CS109 | CS446-1 pHLmob *shyA* | This study |
| MA998 | CS109 | CS446-1 pHLmob *shyA^R115W^* | This study |
| MA1248 | CS109 | pBADmob ShyB | This study |
| MA1252 | CS109 | ∆4 pBADmob ShyB | This study |
| MA902 | CS109 | pBADmob mepM | This study |
| MA904 | CS109 | pBADmob mepM ∆dom1 | This study |
| MA908 | CS109 | ∆4 pBADmob mepM | This study |
| MA910 | CS109 | ∆4 pBADmob mepM ∆dom1 | This study |

References

1. Wilson, A. C., Perego, M. & Hoch, J. A. New transposon delivery plasmids for insertional mutagenesis in Bacillus anthracis. *J. Microbiol. Methods* **71**, 332–335 (2007).
2. Woodcock, D. M. *et al.* Quantitative evaluation of Escherichia coli host strains for tolerance to cytosine methylation in plasmid and phage recombinants. *Nucleic Acids Res.* **17**, 3469–3478 (1989).
3. Ferrières, L. *et al.* Silent mischief: bacteriophage Mu insertions contaminate products of Escherichia coli random mutagenesis performed using suicidal transposon delivery plasmids mobilized by broad-host-range RP4 conjugative machinery. *J. Bacteriol.* **192**, 6418–6427 (2010).
4. Denome, S. A., Elf, P. K., Henderson, T. A., Nelson, D. E. & Young, K. D. Escherichia coli mutants lacking all possible combinations of eight penicillin binding proteins: viability, characteristics, and implications for peptidoglycan synthesis. *J. Bacteriol.* **181**, 3981–3993 (1999).
