# Supplementary material for: Genetic interaction mapping reveals functional relationships between peptidoglycan endopeptidases and carboxypeptidases
Source: PLoS Genet. 2024 Apr 10;20(4):e1011234. doi: 10.1371/journal.pgen.1011234 (PMC11034669; doi:10.1371/journal.pgen.1011234)
Supplement: S6 Table — (DOC) [file pgen.1011234.s020.doc]

**Supplemental Table 6.** **Plasmids used in this study.**

| **Plasmid** | **Description** | **Reference / Source** |
| --- | --- | --- |
| pSC189 | Mariner transposon delivery. | [1] |
| pTD101 | Chromosomal insertion in *lacZ,* IPTG induction. | [2] |
| pTD101 *shyA^L109K^* | ShyA^L109K^ overexpression, whole gene amplification with MA378-MA379 from mutated locus.^9^ | This study |
| pTD101 *zur* | Zur complementation, whole gene amplification with MA616-MA617. | This study |
| pTD101 *shyB* | ShyB overexpression. | [3] |
| pTD101 *shyA* | ShyA overexpression, whole gene amplification with MA378-MA379. | This study |
| pTD101 *shyA^R115W^* | ShyA^L109K^ overexpression, whole gene amplification with MA378-MA379 from suppressor mutant (1S8). | This study |
| pTD101 murA | MurA overexpression, whole gene amplification with MA645-MA646. | This study |
| pTD101 *nlpC* | NlpC overexpression, whole gene amplification with MA691-MA692. | This study |
| pTD101 *tagE1* | TagE1 overexpression, whole gene amplification with MA693-MA694. | This study |
| pTD101 *tagE2* | TagE2 overexpression, whole gene amplification with MA695-MA696. | This study |
| pTD101 *shyC* | ShyC overexpression, whole gene amplification with MA689-MA690. | This study |
| pTD101 VC_A0040 | VC_A0040 overexpression, whole gene amplification with MA649-MA650. | This study |
| pHL100mob | Conjugable derivative of pHL100 | [2] |
| pHLmob *shyA* | ShyA overexpression, whole gene amplification with MA378-MA379. | This study |
| pHLmob *shyA^R115W^* | ShyA^L109K^ overexpression, whole gene amplification with MA378-MA379 from suppressor mutant (1S8). | This study |
| pBADmob | Mobile pBAD33 derivative. |  |
| pBADmob mepM | MepM overexpression. | [4] |
| pBADmob mepM ∆dom1 | MepM ∆dom1 overexpression | [4] |
| pBADmob shyB | ShyB overexpression, whole gene amplification with SM155-SM157a |  |
| pTOX5 | Allele exchange vector | [5] |
| pTOX *dacA1* | Used for deletion of *dacA1* flanking regions were amplified with (upstream) MA354-355 and (downstream) MA356-357. Deletions were validated with flanking (MA358-359) and internal (MA360-361) primers. | This study |
| pTOX *zur* | Used for deletion of *zur* flanking regions were amplified with (upstream) MA405-406 and (downstream) MA407-408. Deletions were validated with flanking (MA464-465) and internal (MA466-467) primers. | This study |
| pTOX *murA^P122S^* | Used for substitution of wt copy of *murA* with *murA^P122S^*, upstream-gene-downstream region was amplified using MA567-568 using gDNA from suppressor mutant (3S7). | This study |
| pTOX *murA^L35F^* | Used for substitution of wt copy of *murA* with *murA^L35F^*, upstream-gene-downstream region was amplified using MA573-574 using gDNA from suppressor mutant (3S8). | This study |
| pTOX *murD^D447E^* | Used for substitution of wt copy of *murD* with *murD^D447E^*, upstream-gene-downstream region was amplified using MA577-578 using gDNA from suppressor mutant (1S1). | This study |
| pET28a | Plasmid to overexpress SUMO-6xHis-tagged MurA protein in E. coli BL21 cells. | Novagen |
| pET28a murA | MurA overexpression, whole gene amplified with MA599-600. | This study |
| pET28a murA^P122S^ | MurA^P122S^ overexpression, whole gene amplified with MA599-600 from suppressor mutant (3S7). | This study |
| pET28a murA^L35F^ | MurA^L35F^ overexpression, whole gene amplified with MA599-600 from suppressor mutant (3S8). | This study |
| pET28a murC | MurC overexpression, whole gene amplified with MA685-686. | This study |
| pET28a murC^A132T^ | MurC overexpression, whole gene amplified with MA685-686 from suppressor mutant (1S3). | This study |

1. Wilson, A. C., Perego, M. & Hoch, J. A. New transposon delivery plasmids for insertional mutagenesis in Bacillus anthracis. *J. Microbiol. Methods* **71**, 332–335 (2007).
2. Cava, F., de Pedro, M. A., Lam, H., Davis, B. M. & Waldor, M. K. Distinct pathways for modification of the bacterial cell wall by non-canonical D-amino acids. *EMBO J.* **30**, 3442–3453 (2011).
3. Murphy, S. G. *et al.* Endopeptidase Regulation as a Novel Function of the Zur-Dependent Zinc Starvation Response. *mBio* **10**, (2019).
4. Shin, J.-H. *et al.* Structural basis of peptidoglycan endopeptidase regulation. *Proc. Natl. Acad. Sci. U. S. A.* **117**, 11692–11702 (2020).
5. Lazarus, J. E. *et al.* A New Suite of Allelic-Exchange Vectors for the Scarless Modification of Proteobacterial Genomes. *Appl. Environ. Microbiol.* **85**, (2019).
