# Supplementary material for: Genetic interaction mapping reveals functional relationships between peptidoglycan endopeptidases and carboxypeptidases
Source: PLoS Genet. 2024 Apr 10;20(4):e1011234. doi: 10.1371/journal.pgen.1011234 (PMC11034669; doi:10.1371/journal.pgen.1011234)
Supplement: S7 Table — (DOC) [file pgen.1011234.s021.doc]

**Supplemental Table 7. Oligonucleotides used in this study.**

| **Primer** | **Sequence 5’ 🡪 3’** | **Description** |
| --- | --- | --- |
| **Deletions** | | |
| MA354 | GGCGGGGTTTTTTCGTTGATCACGTACGATCGATCAAGCACCCGACAC | pTOX-UP-dacA-F |
| MA355 | GACAGTATTGGCTTTACTGGCTGAACGGTTGGTGAATGCTTAGTTCTACTTAAGT | pTOX-UP-dacA-R |
| MA356 | CGGACACTTAAGTAGAACTAAGCATTCACCAACCGTTCAGCCAGTAAA | pTOX-DOWN-dacA-F |
| MA357 | CCTGTACACCATGTGCACCGGTTCGAAGATATCTCCAGTATTGAGGAGGT | pTOX-DOWN-dacA-R |
| MA358 | AGTACAAATAATACGCATCGC | dacA1 colony F |
| MA359 | ACTTTGTAGGTAAAGGAGC | dacA1 colony R |
| MA360 | GGGTAATGATGCGTGTGT | dacA1 colony int F |
| MA361 | TGTCAGTACCCATCACCA | dacA1 colony int R |
| MA405 | GGCGGGGTTTTTTCGTTGATCACGTACGATAACTGAGTTAAAAACTCGTACG | pTOX5 zur UP F |
| MA406 | GTTTACAAATTCAGCGCGCATAACCTTCTTTGTTGGACTTTAGTAATAATATACTATGT | pTOX5 zur UP R |
| MA407 | GACATAGTATATTATTACTAAAGTCCAACAAAGAAGGTTATGCGCG | pTOX5 zur DOWN F |
| MA408 | CCTGTACACCATGTGCACCGGTTCGAAGATCAATGGGATGAGCCGAT | pTOX5 zur DOWN R |
| MA464 | TTTACGGTAAATGGGCGC | zur colony F |
| MA465 | GCATGATTTCCAAAGCAAG | zur colony R |
| MA466 | TAAACGCTCATCAAGTGC | zur int colony F |
| MA467 | GATTCAATCACGTTACCGC | zur int colony R |
| **Chromosomal substitutions** | | |
| MA567 | GGCGGGGTTTTTTCGTTGATCACGTACGATTGGATCTTTCTCAAAAATTGGCA | pTOX-M murA F |
| MA568 | CCTGTACACCATGTGCACCGGTTCGAAGATGAAACATCGCTCTGTTTCATC | pTOX-M murA F |
| MA472 | TCATGTCAAAGGAGAGGGC | murA colony F |
| MA473 | CCGAACACTTTCGACATC | murA colony R |
| MA474 | CGCGCATATTGTGATGGA | murA colony int F |
| MA475 | TTCAAGCTTAGCTAAGACCG | murA int colony R |
| MA573 | GGCGGGGTTTTTTCGTTGATCACGTACGATGCAGCGTTTTGCCACG | pTOX-M murC F |
| MA574 | CCTGTACACCATGTGCACCGGTTCGAAGATTCGATGCATGGGAAACCC | pTOX-M murC R |
| MA460 | GTCTCAGAGGTCTCAGC | murC colony F |
| MA461 | GTGATTCTCTGGCCTTCT | murC colony R |
| MA462 | GGATCCGACCTTTGTGA | murC int colony F |
| MA463 | CGTAAAATCGCTTCATCG | murC int colony R |
| MA577 | GGCGGGGTTTTTTCGTTGATCACGTACGATTACATTGTGCTGACCTATTTCG | pTOX-M murD F |
| MA578 | CCTGTACACCATGTGCACCGGTTCGAAGATTAGGTTTTAAAAAGCCGCCA | pTOX-M murD R |
| MA456 | GTATTTATGGGCGATGTCG | murD colony F |
| MA457 | CAACAGTAGAAAGATGGCG | murD colony R |
| MA458 | TTAGCCAATGCTGCTGG | murD int colony F |
| MA459 | CTAACACGGTAAGTACGTTT | murD int colony R |
| **Overexpression in *V. cholerae*** | | |
| MA476 | GGCAAATATTCTGAAATGAGCTGT | pTD101 colony F |
| MA477 | CCAGATCTTAATTAAGGTGCGTTCT | pTD101 colony R |
| MA377 | AACAGACCATGGAATTCGAGCTCGGTACCCAGGAGGCTGACTGAGTGATATCTAAATCTATTATTTTGCGATT | pTD101 RBS shyA F |
| MA378 | ATGCCTGCAGGTCGACTCTAGAGGATCCCCTTATTGCGCTGCTAGCAT | pTD101 shyA R |
| MA616 | AACAGACCATGGAATTCGAGCTCGGTACCCAGGAGGCTGACTGAGTGATGGTGATGGTTTTGGAC | pTD101 rbs zur F |
| MA617 | ATGCCTGCAGGTCGACTCTAGAGGATCCCCCTATTGTTGCTTCTTCTCTTTCGAG | pTD101 zur R |
| MA620 | AACAGACCATGGAATTCGAGCTCGGTACCCAGGAGGCTGACTGATTGGCAGGAAAAACAAT | pTD101 RBS VCA0040 F |
| MA621 | ATGCCTGCAGGTCGACTCTAGAGGATCCCCCTACTTTTCGGCGTATTTTTCTAAACC | pTD101 VCA0040 R |
| MA645 | AACAGACCATGGAATTCGAGCTCGGTACCCAGGAGGCTGACTGAATGGAAAAGTTTCGAGTTATTGG | pTD101-murA F |
| MA646 | ATGCCTGCAGGTCGACTCTAGAGGATCCCCCTAGTCGCGGAAACGC | pTD101-murA R |
| MA647 | AACAGACCATGGAATTCGAGCTCGGTACCCAGGAGGCTGACTGAATGCTGACAAGGTGGTG | pTD101-murC F |
| MA648 | ATGCCTGCAGGTCGACTCTAGAGGATCCCCTTAAATCTGTTGCATACGCCC | pTD101-murC R |
| MA649 | AACAGACCATGGAATTCGAGCTCGGTACCCAGGAGGCTGACTGATTGGATCATCTCTATCGTTTTAG | pTD101-murD F |
| MA650 | ATGCCTGCAGGTCGACTCTAGAGGATCCCCTAAAGCTTATGCATATTGCCGA | pTD101-murD R |
| MA689 | AACAGACCATGGAATTCGAGCTCGGTACCCAGGAGGCTGACTGAATGCTTTCTCTTTTCAATCGTC | pTD101-RBS-shyC-F |
| MA690 | ATGCCTGCAGGTCGACTCTAGAGGATCCCCTTATTGATTGGCATACAGTAACTG | pTD101-shyC-R |
| MA691 | AACAGACCATGGAATTCGAGCTCGGTACCCAGGAGGCTGACTGAATGTTGACGGTCAAGCAA | pTD101-RBS-nlpC-F |
| MA692 | ATGCCTGCAGGTCGACTCTAGAGGATCCCCTTAGTTTGACAACAAAGGCG | pTD101-nlpC-R |
| MA693 | AACAGACCATGGAATTCGAGCTCGGTACCCAGGAGGCTGACTGAATGAATAAGACACTTAGGATTACATT | pTD101-RBS-tagE1-F |
| MA694 | ATGCCTGCAGGTCGACTCTAGAGGATCCCCTTATTGTGCATTGAGATTCGG | pTD101-tagE1-R |
| MA695 | AACAGACCATGGAATTCGAGCTCGGTACCCAGGAGGCTGACTGAATGAAAGAAAGAATGATTGTTTCTGT | pTD101-RBS-tagE2-F |
| MA696 | ATGCCTGCAGGTCGACTCTAGAGGATCCCCTTAACTCTCCGGAAAAATGGC | pTD101-tagE2-R |
| MA106 | CATGATTGGCATGGTGGAGC | LacZ Flank F |
| MA107 | GTATTGCACAGGACGCGATG | LacZ Flank R |
| **Overexpression in *E. coli*** | | |
| MA377 | AACAGACCATGGAATTCGAGCTCGGTACCCAGGAGGCTGACTGAGTGATATCTAAATCTATTATTTTGCGATT | pHL100mob RBS shyA F |
| MA378 | ATGCCTGCAGGTCGACTCTAGAGGATCCCCTTATTGCGCTGCTAGCAT | pHL100mob shyA R |
| SM44b | CTGTTGACAATTAATCATCCGGCTCG | pHL100mob colony F |
| SM45b | ACTGCCGCCAGGCAAATTC | pHL100mob colony R |
| SM155 | ATGCCTGCAGGTCGACTCTAGAGGATCCCCGCTATTTTTATTAAGTTTCGTCGAGAGCAAC | ShyB-pBAD-R |
| SM157a | TTTGGGCTAGCGAATTCGAGCTCGGTACCCAGGAGGCTGACTGAATGGGTCAATTTAGATTTCTTGCTTTAATCG | ShyB-pBAD-F |
| SM42 | GCAACTCTCTACTGTTTCTCCATACC | pBAD33-F |
| SM43 | GCGTTCTGATTTAATCTGTATCAGGC | pBAD33-R |
| **Protein Purification** | | |
| MA599 | AGGCTCACAGAGAACAGATTGGTGGATCCGAAGAAAAGTTTCGAGTTATTGGGTCC | pET28a-HIS-SUMO-MurA-F |
| MA600 | GTGCGGCCGCAAGCTTGTCGACGGAGCTCGCTAGTCGCGGAAACGCTC | pET28a-HIS-SUMO-MurA-R |
| MA685 | AGGCTCACAGAGAACAGATTGGTGGATCCGAACTGACAAGGTGGTGGC | pET28a murC F |
| MA686 | GTGCGGCCGCAAGCTTGTCGACGGAGCTCGTTAAATCTGTTGCATACG | pET28a murC R |
