# Supplementary material for: Genetic interaction mapping reveals functional relationships between peptidoglycan endopeptidases and carboxypeptidases
Source: PLoS Genet. 2024 Apr 10;20(4):e1011234. doi: 10.1371/journal.pgen.1011234 (PMC11034669; doi:10.1371/journal.pgen.1011234)
Supplement: Attachment — Submitted filename: Review_PLOS_Genetics.docx [file pgen.1011234.s022.docx]

The paper from Alvarado Obando et al. employs Tn-Seq in *Vibrio cholerae* encoding a hyperactive endopeptidase background (*shyA^L109K^*) to find new regulators for endopeptidase activity*.* Critically, the authors identified the carboxypeptidase dacA1 (PBP5) as a so far unknown inhibitor of endopeptidase mediated cell wall degradation as shown through *in vitro* PG degradation experiments suggesting that elevated penta-peptide cross-links are unable to be efficiently processed by ShyA.

Interestingly, DacA1 itself is essential for growth in environments containing salt (such as LB) and cells lacking it display sever growth defects. Thus, a dcaA1 deletion is only advantageous in a context of hyperactivated endopeptidase activity, therefore ∆dacA1 is suppressed by mutants with elevated endopeptidase activity. Overall, this suggests that shyA (endopeptidase) and dacA1 (carboxypeptidase) are genetically linked. This interpretation is further supported by the fact that an overexpression of ShyA also alleviates the morphological defects of an E. coli strain lacking all 4 carboxypeptidases.

An alternative set of ∆dacA1 suppressor could restore growth, but not morphology, through a series of hypermorphic mutations in PG precursor synthesis, which are believed to result in reduced cell wall synthesis rates. The salt-sensitivity of ∆dacA1 might be linked to a reduced undecaprenol pyrophosphate translocation rate, which was shown to be dependent on the sodium motive force. However, it remains unclear how reduced PG synthesis rates mediate the suppression of ∆dacA1 in salt-containing media.

Alvarado Obando and colleagues address an important aspect of cell wall biogenesis, namely how carboxypeptidase ensure the balance between PG synthesis and hydrolysis, which is currently poorly understood. This work lays the foundation for more intricate studies following up on the molecular mechanism and the spatiotemporal regulation of this mechanism. Overall, the paper is well written and the first part on of the manuscript is technically sound and all major conclusions are supported by experimental data. However, prior to publication the authors should address the point outlined below:

Major comments:

- The manuscript lacks data on protein levels, although several phenotypes are attributed to changes in protein levels (e.g.: LL. 176-187 ShyA/B/ShyA^R115W^). Similarly, knowing protein levels and stability in Mur suppressors (LL. 234-235 MurA^L35F^) would be informative to better understand the mechanism of suppression (in particular murC, murD which lack enzymatic assay).
- All growth defects are assessed from CFU assays. I believe the manuscript would benefit from showing growth curve data, particularly for some of the weaker ∆dacA1 suppressors, whose effect might be stronger in exponential phase as compared to stationary phase (e.g. Mur suppressors, particularly ,murD^D447E^ has a small colony size phenotype, ∆VCA0040).
- The main finding of this paper is revealing the unknown functional relationship between carboxypeptidases and endopeptidases and how both activities are needed to mediate proper cell growth and morphology (Fig. 1-4, Fig. 7). In my opinion the finding that reduced cell wall synthesis rescues a carboxypeptidase defect in salty environments to me is not adding much to the main point of the story and rather distracting my attention. It is not too surprising or novel that a reduction in growth rate is promoting the survival of cells suffering from PG defects and overall the phenotypes are much weaker than for the enhanced endopeptidase activity suppressors.
- More importantly, the most prominent class of *∆dacA1* suppressors is found in genes in OM and O-antigen biogenesis (e.g. WaaL) (Table S2). Why is this not mentioned in the text? The importance of the mechanical properties of the OM in mediating cell envelope integrity and morphology have gained recent interest (Fivenson et al 2023, Rojas 2018) and would merit more thorough investigation and discussion.

Minor comments:

Introduction:

- LL65-66: This statement should be referenced.
- LL79: Referenced not continuously -> (6-8)
- LL86-96: These statements should be referenced.
- Figure 1: The non-expert reader would benefit from a simple cartoon in Fig. 1. showing the sites of PG cleavage on the cell wall.

Results

- LL121, 129, 172, 189, 219: ‘To answer the screen…’ Maybe this is my non-native speaker background, but I am not a huge fan of this expression. I would go with something like ‘revealed/resulted in/found mutations in XY’
- LL127: YhdP is specifically mentioned, but not highlighted on the graph (Fig. 1a) Please highlight or not mention.
- LL164-166: There are no quantification of salt-free morphologies, and it is not discussed why ShyA^L109K^ overexpression is not restoring wt morphology? Please elaborate on this. Also in my opinion area measurements are the least informative measure of cell shape. Length and width are more insightful. For example, it seems that in salt-free media ∆dacA1 are not only longer, but also wider. This might highlight a reduced SEDS/bPBP cell wall synthase activity when pentapeptide levels are elevated, which can be compensated by increasing aPBPs activity. This could go also into discussion LL359-360.
- LL213-216: Could the authors please elaborate more on (i) what D44 and D45 refers to and (ii) why it is so important that endopeptidase hydrolyze pentapeptode-containing crosslinks at low efficiency?
- LL224: The authors could attempt to measure PG synthesis rates e.g. using metabolic incorporation of biorthogonal MurNAc (Liang et al. 2017) or D-Ala-D-Ala (Liechti et al. 2014) probes, or radiolabeled mDAP, to corroborate reduced PG synthesis rates in Mur suppressors and ∆VCA0040, or at least explain why this is not possible/needed (e.g. by showing growth curves).
- LL253-256: Please add reference to Fig. 5c.
- LL 281: It would be great if the authors could show a quantification of recovered CFUs over multiple repeats or show additional plates in supplementary figures.
- LL296-300: The deletion of VCA0040 only provides a minor growth advantage, often there are no visible single colonies on the last dilution step (FigS10). As above, quantification would help.
- LL304: Why do the authors not use the MurA construct to make a depletion strain to buttress their findings on how growth rate reduction provides survival in ∆dacA1?
- LL309: Again, please provide quantification of recovered CFUs from multiple replicates.
- LL316-325: Does this strain in addition to its impressive morphological defects also have a growth defect? If yes, does ShyA expression restore this? Moreover, it seems that even in presence of glucose ShyA is potently repressing the growth defects in the ∆4 strain. This should be discussed.

Discussion:

- LL372-374: I am not fully convinced by the provided data that the authors can unambiguously state that these mutation result in reduced PG synthesis rates. Please address some of my point above.

Methods:

- LL414-417: There are no growth curves in this manuscript. Please add to address my concerns above.
- LL420-421: 1mM IPTG induction seems like an overkill. Again knowing protein levels would tremendously help to understand if these experiments are physiologically meaningful.

Figures:

- Fig 1: add cartoon of PG hydrolase activity
- Fig. 2: c) Since lysozyme is used as a pos. control change endopeptidase activity to hydrolase activity
- Fig. 3: b)Please provide morphological quantification for SF phenotypes here or in S1. C) exchange with length width data from S1.
- Fig. 5: c) The i in inorganic phosphate (P_i_) should be subscript as it might be confused with propidium iodide
