# Supplementary material for: Genetic interaction mapping reveals functional relationships between peptidoglycan endopeptidases and carboxypeptidases
Source: PLoS Genet. 2024 Apr 10;20(4):e1011234. doi: 10.1371/journal.pgen.1011234 (PMC11034669; doi:10.1371/journal.pgen.1011234)
Supplement: Attachment — Submitted filename: Response to reviewers.docx [file pgen.1011234.s023.docx]

**Response to reviewers**

We thank all reviewers for a thorough and constructive reading of our manuscript. Please find below our response to specific comments.

**Reviewer 1**

Major comments

R1: It seems plausible that ShyA activity itself is salt-sensitive – and that it is more active at low salt. Have the authors tested this?

We think that this is very much a possibility, and we are in fact currently preparing a separate manuscript suggesting this. However, our conclusions here are not dependent on whether or not ShyA is more active in low salt, and this represents a whole research direction by itself.

R1: What is the activity in Fig. 2C relative to – none of the bars matches ‘1’ so this is hard to see?

Figure 2C has been updated to show the raw data instead of the normalized data. It shows the absorbance values for the samples as well as the negative control (reaction buffer) for easier interpretation.

R1: Also, if L109K is a hyperactive mutant, why is there no discernable difference in the in vitro activity of ShyA and ShyAL109K on wildtype sacculi? Is the assay saturated.

The assay is in fact saturated, the data presented is from a 24h reaction and 0.8 is the highest relative absorbance that can be detected. This is expected since we know that ShyA’s activity is temporal and even though the WT ShyA has a lower activity during the initial few minutes to hours of the reaction, its activity reaches the same level as ShyA^L109K^ after 20h (1). We thought that overdigestion rather than underdigestion would most rigorously test the hypothesis that ∆*dacA1* PG is more resistant against EP cleavage.

R1: What happens to the number of crosslinks in the wildtype when grown on high salt (I assume all results in TS3 are low salt since the dDacA1 cannot grow on high salt)?

The PG profiling was performed in LB media, not SFLB. Though ultimately the mutant fails to form colonies on a plate, mass growth of the *∆dacA1* mutant in liquid medium is sufficient to extract and analyze PG samples. WT crosslinks in LB medium (180 mM NaCl) are presented in Table S3; however, crosslinks weren’t measured in higher concentrations of NaCl.

R1: I would expect a more thorough investigation of the effects of salt on the activities of both DacA1 and ShyA (mutants).

These experiments would be nice but are not straight-forward. As stated above, our conclusions do not rely on whether the activities of ShyA or DacA1 vary with salt concentrations; however, we have now added this as a possible explanation of the dacA1 mutant’s salt sensitivity to the discussion section. We have some unpublished data suggesting that at least ShyA might be more active under conditions of high turgor (e.g., in low salt), but these data are part of a separate line of inquiry (manuscript in preparation). At any rate, we believe that this is outside of the scope of this paper, which focuses on the genetic relationship between endopeptidases and carboxypeptidases. Salt sensitivity just happens to be the phenotypic tool we employ here to probe this relationship.

Minor comments

- The authors should specify growth conditions for experiments in all figure or table legends given the importance of the presence/absence of salts for the phenotypes.

Growth conditions have been added to the figure legends.

- The introduction is phrased very ‘general’ – as if the statements made are valid for all bacteria. This is not the case; the authors should be more specific. Examples:
  L 47-50: description of PG, stem peptide composition, is correct for typical Gram-negative PG but reads as if valid for all bacteria.

This section has been modified to specify that the side peptide composition is specific to *V. cholerae*.

- L 66: pentapeptide is not “exclusively” associated with newly synthesized PG – eg Neisseria meningitidis has relatively high levels of pentapeptide in its PG (<https://doi.org/10.1371/journal.pgen.1005338>) and B. subtilis had pentapeptides enriched at division sites (<https://doi.org/10.1111/mmi.13629>).

The reviewer raises a valid point. What we meant was that pentapeptide exclusively originates from nascent PG (only crosslinking of two sidestems that both contain pentapeptide results in retention of pentapeptide in one of them). Whether or not the pentapeptide then persists in “older” PG depends on the activity of carboxypeptidases. We rephrased the statement in the manuscript to reflect this.

- L 71 DacA not DacA1. Edited
- L112 if ShyAL109K is already hyperactive, why does it need to be overexpressed as well?

We do not think that it necessarily has to be overexpressed (compare with R115W from our *dacA1* suppressor screen; this mutation in ShyA rescues the phenotype in single copy on the chromosome). This just happens to be how we started the TnSeq experiment.

- L 114 – ref 12 is preprint of ref 11- Citation corrected
- L 121 and further – the use of the phrase ‘the screen was answered’ – I find this an odd turn of phrase and don’t recall reading it elsewhere in papers using TnSeq. Matter of taste but I would rephrase.

We rephrased throughout.

- L 213-4 D44 and D45 should be explained

It is now explained in the text.

- L 224 – “hypomorph” – I don’t know what is meant by this expression.

A hypomorph is an allele with reduced function.

- L349-50 “tetrapeptide and can only serve as donor” – shouldn’t this be “acceptor”?

This is correct, this has been fixed in the manuscript.

**Reviewer 2:**

Major comments

R2: The manuscript lacks data on protein levels, although several phenotypes are attributed to changes in protein levels (e.g.: LL. 176-187 ShyA/B/ShyA^R115W^). Similarly, knowing protein levels and stability in Mur suppressors (LL. 234-235 MurA^L35F^) would be informative to better understand the mechanism of suppression (in particular murC, murD which lack enzymatic assay).

We agree that this would be informative. We do possess antibodies against ShyA and have quantified EP levels via Western Blot (now **Fig S1D**). L109K levels (but not those of R115W) are elevated in the WT, but not in the *dacA1* mutant (see also comment below in response to Reviewer 3). We do not understand the reason for this difference (ShyA regulation is complex and relies partially on degradation by two periplasmic proteases); however, the observation that R115W rescues ∆*dacA1* defects while displaying wt levels suggests that an increase in EP abundance alone is not sufficient for suppression. We do not have antibodies specific to MurA, MurC and MurD, and chromosomal epitope tagging may affect function. However, our conclusions regarding the mur mutants rest on two pieces of orthogonal data: We demonstrate reduced enzymatic activity *in vitro* at least for MurA, and partial inhibition of MurA (by Fosfomycin) rescues ∆*dacA1* phenotypes.

We were also able to purify MurC and MurC^A132T^ and measure their enzymatic activity with a commercial kit. We found that MurC^A132T^ has significantly lower activity than WT MurC, providing additional evidence that this mutant reduces the activity MurC and consequently also reduces the rate of PG synthesis. Due to technical difficulties, we were unable to measure MurD and MurD^D447E^ activities. These results can now be found on **Fig. 5D.**

R2: All growth defects are assessed from CFU assays. I believe the manuscript would benefit from showing growth curve data, particularly for some of the weaker ∆dacA1 suppressors, whose effect might be stronger in exponential phase as compared to stationary phase (e.g. Mur suppressors, particularly, murD^D447E^ has a small colony size phenotype, ∆VCA0040).

Thank you for the suggestion, we performed these experiments, and they are now part of **Fig. S7A** and **Fig. S12B**. These data further demonstrates that the suppressor mutations in murA, murC and murD confer a (albeit subtle) growth advantage in LB, which is more evident during exponential phase.

R2: The main finding of this paper is revealing the unknown functional relationship between carboxypeptidases and endopeptidases and how both activities are needed to mediate proper cell growth and morphology (Fig. 1-4, Fig. 7). In my opinion the finding that reduced cell wall synthesis rescues a carboxypeptidase defect in salty environments to me is not adding much to the main point of the story and rather distracting my attention. It is not too surprising or novel that a reduction in growth rate is promoting the survival of cells suffering from PG defects and overall the phenotypes are much weaker than for the enhanced endopeptidase activity suppressors.

From the growth curves done with the *mur* mutants in both backgrounds we can conclude that the effect of these mutations does not result in a reduction in growth rate. The *mur* mutations in the WT background maintain the same growth rate as WT. This, in addition to the MurA and MurC enzymatic assays suggest that growth rate reduction is not what allows growth of the ∆*dacA1* mutant but rather the reduction in PG synthesis or increase in PG cleavage.

R2: More importantly, the most prominent class of ∆dacA1 suppressors is found in genes in OM and O-antigen biogenesis (e.g. WaaL) (Table S2). Why is this not mentioned in the text? The importance of the mechanical properties of the OM in mediating cell envelope integrity and morphology have gained recent interest (Fivenson et al 2023, Rojas 2018) and would merit more thorough investigation and discussion.

We were also very intrigued by the suppressor mutants that mapped to genes involved in LPS biogenesis. Our initial hypothesis was that these mutants, especially *waaL* would result in precursor sequestration of Und-P (2) that would reduce PG synthesis due to hindered recycling of the lipid carrier. However, we realized that even though VC_0393 is annotated as O-antigen ligase, it is not *waaL* but actually a homolog of *pglL*, a predicted oligosaccharyltranferase involved in protein glycosylation. Its involvement in PG homeostasis thus remains a mystery for now, but we will explore this in future work. Annotation has been corrected on **Supplemental Table 2**.

Minor comments

Minor comments:

Introduction:

- LL65-66: This statement should be referenced. Reference added
- LL79: Referenced not continuously -> (6-8) Edited
- LL86-96: These statements should be referenced. Edited
- Figure 1: The non-expert reader would benefit from a simple cartoon in Fig. 1. showing the sites of PG cleavage on the cell wall. Now **Fig 1A.**

Results

- LL121, 129, 172, 189, 219: ‘To answer the screen…’ Maybe this is my non-native speaker background, but I am not a huge fan of this expression. I would go with something like ‘revealed/resulted in/found mutations in XY’

We rephrased throughout.

- LL127: YhdP is specifically mentioned, but not highlighted on the graph (Fig. 1a) Please highlight or not mention.

It is now highlighted on the graph (Now **Fig 1B**)

- LL164-166: There are no quantification of salt-free morphologies, and it is not discussed why ShyA^L109K^ overexpression is not restoring wt morphology? Please elaborate on this. Also in my opinion area measurements are the least informative measure of cell shape. Length and width are more insightful. For example, it seems that in salt-free media ∆dacA1 are not only longer, but also wider. This might highlight a reduced SEDS/bPBP cell wall synthase activity when pentapeptide levels are elevated, which can be compensated by increasing aPBPs activity. This could go also into discussion LL359-360.

Length and width measurements (instead of area measurements) have now been added to **Figure 3**. Regarding length and width measurements in salt-free LB, we in principle agree with the reviewer that these are superior in most situations. However, our cells have such aberrant morphologies, that length and width can be difficult to measure (especially in those cells overexpressing ShyA^L109K^, which causes spheroplast formation, blebbing and lysis in many cells). However, following the reviewer’s suggestion, we compared the differences in length and width between WT and *∆dacA1,* finding that when grown in salt-free LB *∆dacA1* cells are in fact longer and wider than WT (**Fig. S1C**) We have also added a discussion of the possible contribution of SEDS vs. aPBPs as possible underlying cause for the *dacA1* phenotypes.

- LL213-216: Could the authors please elaborate more on (i) what D44 and D45 refers to and (ii) why it is so important that endopeptidase hydrolyze pentapeptide-containing crosslinks at low efficiency?

We have added a description of what D44 and D45 refers to and added text to this section more clearly outlining the significance of this observation.

- LL224: The authors could attempt to measure PG synthesis rates e.g. using metabolic incorporation of biorthogonal MurNAc (Liang et al. 2017) or D-Ala-D-Ala (Liechti et al. 2014) probes, or radiolabeled mDAP, to corroborate reduced PG synthesis rates in Mur suppressors and ∆VCA0040, or at least explain why this is not possible/needed (e.g. by showing growth curves).

Though of course highly informative and interesting, there are unfortunately significant challenges associated with the proposed methods. (1) NAM labelling requires the addition of Fosfomycin to ensure the incorporation of the labelled NAM, our data shows that Fosfomycin rescues *∆dacA1* so this type of labelling wouldn’t recapitulate the ∆dacA1 phenotype. (2) D-ala-D-ala labelling would give us an imprecise quantification as well since D-ala-D-ala is the substrate of DacA1, in the ∆*dacA1* mutant we would have increased levels of the fluorescent D-amino acids, making it difficult to compare to WT. (3) Since we are working with a non-model organism (*V. cholerae)*, the assays using radiolabeled mDAP are not established, and our lab lacks the expertise to do them. Following the reviewer’s suggestions, we have instead added growth curve experiments to more quantitatively show the effects caused by the suppressor mutation.

- LL253-256: Please add reference to Fig. 5c (Now Figure 5D).

Thanks for pointing this out, it is now referenced in the text.

- LL 281: It would be great if the authors could show a quantification of recovered CFUs over multiple repeats or show additional plates in supplementary figures. (antibiotics)

We have now included pictures of additional replicates that reiterate the subtle phenotype (**Fig. S10-S11**).

- LL296-300: The deletion of VCA0040 only provides a minor growth advantage, often there are no visible single colonies on the last dilution step (FigS10). As above, quantification would help.

Pictures of additional replicates as well as growth curves that show deletion of *vca0040* confers a growth advantage to ∆dacA1 have been added to **Fig S12B**.

- LL304: Why do the authors not use the MurA construct to make a depletion strain to buttress their findings on how growth rate reduction provides survival in ∆dacA1?

We do not think that it is growth rate reduction (see growth curves above for Mur suppressors). Please note that we have essentially done this experiment, albeit with chemical inactivation (Fosfomycin) rather than depletion.

- LL309: Again, please provide quantification of recovered CFUs from multiple replicates. (Salt sensitivity)

Pictures of additional replicates have been added to **Fig S13**.

- LL316-325: Does this strain in addition to its impressive morphological defects also have a growth defect? If yes, does ShyA expression restore this? Moreover, it seems that even in presence of glucose ShyA is potently repressing the growth defects in the ∆4 strain. This should be discussed.
- The ∆4 strain does present a slight growth defect that can be rescued by ShyA overexpression as shown in **Fig S4A**. The P_IPTG_ promoter used to express ShyA is a lac promoter, which is known to have leaky expression (even in the presence of glucose) (3).

Discussion:

- LL372-374: I am not fully convinced by the provided data that the authors can unambiguously state that these mutation result in reduced PG synthesis rates. Please address some of my point above.

We base our conclusions on two pieces of orthogonal data: Our biochemical assays demonstrate that at least the MurA and MurC alleles have reduced activity in vitro, and our chemical complementation assays reveal that ∆*dacA1* phenotypes can be rescued by addition of Fosfomycin (a well-established inhibitor of MurA).

Methods:

- LL414-417: There are no growth curves in this manuscript. Please add to address my concerns above. Edited
- LL420-421: 1mM IPTG induction seems like an overkill. Again knowing protein levels would tremendously help to understand if these experiments are physiologically meaningful. Edited

Figures:

- Fig 1: add cartoon of PG hydrolase activity. Added to Fig 1
- Fig. 2: c) Since lysozyme is used as a pos. control change endopeptidase activity to hydrolase activity. Edited
- Fig. 3: b) Please provide morphological quantification for SF phenotypes here or in S1. We quantified Length and Width of WT and ∆dacA1 cells grown in salt-free LB C) exchange with length width data from S1. Edited
- Fig. 5: c) The i in inorganic phosphate (P_i_) should be subscript as it might be confused with propidium iodide. Edited

**Reviewer #3:**

Major points:

R3: Lane 130-136: How does the Tn-seq profile look for the other genes encoding enzymes with carboxypeptidase activity (dacA-2, dacB and pbpG)? As they have little influence on the percentage of pentapeptides in the PG matrix in these conditions, they should not help to tolerate ShyAL109K toxicity.

As the reviewer suggests, *dacA2* and *pbpG* do not have significant fold changes meaning they are not required for tolerance of ShyA^L109K^ toxicity. On the other hand, *dacB* (also known as PBP4) came up as conditionally essential. However, since this enzyme carries carboxypeptidase and endopeptidase activity, we cannot conclusively attribute its conditional essentiality to pentapeptide content.

Lane 183-187: Overexpression of ShyB can suppress ∆dacA-1 growth defect. Is ShyB activity affected by the presence of the pentapeptide content in the PG matrix, like in the case of ShyA? *V. cholerae* genome encode many additional endopeptidases. Can any of them suppress *∆dacA-1* defects?

Substrate selectivity of ShyB has not been determined. However, its overexpression without activation is sufficient to rescue the ∆dacA1 phenotype, unlike ShyA, perhaps suggesting that pentapeptide content does not affect the endopeptidase activity of WT ShyB, or that ShyB is intrinsically more active than ShyA (and hence transcriptionally regulated, rather than constitutive).

We overexpressed the remaining 4 D,D-endopeptidases encoded in *V. cholerae* genome (*nlpC,* *tagE1*, *tagE2* and *shyC*) and found that overexpression of tagE2 and shyC rescues the morphological defects caused by *dacA1* deletion, which further supports the idea that the *∆dacA1* morphological defects are due to increased pentapeptide content impeding EP-mediated cleavage of crosslinks. These results are now presented in **Fig. S3**.

Lane 195-197: What is the level of the ShyA, ShyAR115W and ShyAL109K proteins. Could the difference in toxicity in the WT and ability to rescue growth and morphology in the ∆dacA-1 depends on the protein level or protein stability?

Western blot is now included in **Fig S1D**. Protein levels of ShyAL109K do appear to be elevated in the ∆dacA1 background suggesting that higher protein stability in addition to increased enzymatic activity may rescue the ∆dacA1 phenotype when this mutant is overexpressed. However, ShyAR115W presented protein levels similar to those of WT, indicating that protein stability is not what rescues ∆*dacA1* but rather increased enzymatic activity of this mutant.

Lane 232-233: Is the murA::murAL35F strain more sensitive to Fosfomycin? As it has reduced activity (Fig. 5C), but the WT with this mutation seems to be perfectly happy (no shape or plating defects), we might at least expect a higher sensitivity to Fosfomycin.

Thanks for the suggestion, we performed 3 independent antibiotic susceptibility assays with discs containing 10µL of Fosfomycin (50 mg/mL). This experiment revealed that the *murA* mutations do increase Fosfomycin sensitivity in both WT and *∆dacA1* backgrounds, probably due to their reduced basal activity. This data has now been added to **Fig S7**.

Lane 236-237: Is there a specific reason why the authors did not reconstitute the murCA132T mutation in the WT and ∆dacA-1 strain? Were there any technical issues?

We faced technical issues in the early stages of cloning, plasmid construction in *E. coli* of this mutant wasn’t possible in either background. We are not sure of the reasons for this, but it is conceivable that *V. cholerae* MurC^A132T^ is toxic in *E. coli*.

Lane 297-300: How does the double mutant ∆dacA-1∆vca0040 cells look like? As this mutation should impact PG synthesis by affecting the level of PG precursor, we might expect a phenotype similar to the murA, murC or murD mutations and no improvement of the cell morphology.

According to the reviewer’s suggestions, we now imaged the cells. Deleting *vca0040* in the *dacA1* background does improve the cell morphology of *∆dacA1* (**Fig S12**), however not to the level of endopeptidase overexpression.

Lane 304-305: The cited paper (Hernández et al., 2020) used a Pbad plasmid to overexpress murA, which is quite different from the single integration used by the authors here. The effect of murA overexpression might be stronger if murA was expressed from a similar replicative plasmid.

*Vibrio cholerae* has defense systems that eliminate plasmids, notably pBAD (4); for this reason we opted for chromosomal insertions for stable expression. As the reviewer correctly points out, this poses a caveat of reduced expression, but we believe this system is closer to physiological conditions.

Lane 323-325: Have you tried to overexpress shyB or an E. coli endopeptidase to correct the ∆4 defects? In the cited paper, Laubacher et al ., 2013 did not characterize the endopeptidases mutations that correct the morphological defects of the strains lacking 3 or 4 PBPs. It would be a nice addition to show that overexpression of E. coli own endopeptidases does indeed correct the defect of this ∆4 strain, as suggested but not confirmed by this previous study. Such results would strengthens that the role of endopeptidases in correcting carboxypeptidase morphological defects is conserved.

We performed the suggested experiment by overexpressing ShyB, as well as a known ShyA homolog in E. coli, MepM. We overexpressed both the wild-type (WT) form and a mutant lacking Domain 1 (MepM ∆dom1), which is known to have higher activity (1). We found that expression of WT MepM in the ∆4 strain did not alleviate the morphological defects, but overexpression of MepM ∆dom1 did, which suggests that MepM, just like ShyA in *V. cholerae*, needs to be activated to rescue the carboxypeptidase deficiency. Overexpression of MepM ∆dom1 proved to be toxic in both WT and ∆4; however, it seems like leaky expression when grown in glucose is sufficient to rescue the morphological defects as well as the slight growth defect. Intriguingly, overexpression of ShyB resulted in filamentation, which may be explained by previous findings of endopeptidases antagonizing cell division (5). These results provide more evidence supporting the hypothesis that endopeptidases can also rescue carboxypeptidase deficiency in *E. coli.* These results can be found in **Fig S14**.

Figure 3, Figure 4, Figure 6B, Supplemental figure 6-10-11: Do the WT and ∆dacA-1 strain harbor the PIPTG-empty insertion at the lacZ locus? Should not these strains be used for proper comparison? Especially since the effects are quite small (see Figure 6B, suppl. Figure 10-11), we should rule out that the insertion might be responsible for the mild effect observed.

All the experiments that use the P_IPTG_ expression from the lacZ locus have now been repeated with an P_IPTG_-empty insertion in WT and ∆*dacA1* as a control. (Figures can be found in **Supplemental Figures 1B, 2D, 12A and 13B**). Insertion in the lacZ locus did not change our results in either background.

Minor points:

- Lane 212-213-214: For a broad audience who is not necessarily familiar with the in vivo PG analysis, the abbreviations M5-D44-D45 should be explained or illustrated. It is now explained in the text.
- Lane 224: “The mutations in MurA and MurC…” MurA should be replaced by MurD. Edited
- Chapter starting at lane 313: Please, keep the same name all along the text for the E. coli strain: ∆4 or∆4Ec. Edited
- Lane 452: flanking instead of flaking primers. Edited
- Lane 531: SF, probably Salt Free but the abbreviation has not been introduced previously. Edited
- Lane 563: enolpyruvate instead of enolpyrupate. Edited
- Lane 565: Precise (Fig. 5C). Edited
- Lane 745: two-way ANOVA instead of 2way ANOVA. Edited
- Lane 761-762: Domain 1 and Domain 3 instead of Dom1 and Dom3. Edited
- Lane 769-770: Please, adjust the italic and add “with”: “replacements of murA and murD with murAP122S, murAL35F and murDD447E, respectively”. Edited
- Lane 791: “1 mM or 0.2% glucose”, IPTG is missing. Edited
- Figure 3A: Like previously, does SF stand for Salt Free? If yes, please introduce the abbreviation somewhere. All the end of the legend can be removed as it is not relevant for this figure.

Edited per the reviewer’s suggestion.

- Figure 3B: Please, indicate the size represented by the bar. Edited
- Figure 5E: Please, increase the size of the text under the size bar. Edited
- Supplemental figure 1 legend: MicrobeJ instead of MicobeJ. Edited
- Supplemental figure 3 legend lane 8: 1 mM IPTG or (instead of ot) 0.2% glucose. Edited
- Supplemental figure 4: What the role of the GSK motif? It was never introduced. Edited
- Supplemental figure 6 legend: MurCA132T instead of MurAA132T. Modify the name of the strain in the figure: WT PIPTG-MurCA132T instead of MurCA123T. Edited

1.	Shin JH, Sulpizio AG, Kelley A, Alvarez L, Murphy SG, Fan L, et al. Structural basis of peptidoglycan endopeptidase regulation. Proc Natl Acad Sci U S A. 2020 May 26;117(21):11692–702.

2.	Jorgenson MA, Young KD. Interrupting Biosynthesis of O Antigen or the Lipopolysaccharide Core Produces Morphological Defects in Escherichia coli by Sequestering Undecaprenyl Phosphate. Gourse RL, editor. J Bacteriol. 2016 Nov 15;198(22):3070–9.

3.	Nielsen BL, Willis VC, Lin CY. Western blot analysis to illustrate relative control levels of the lac and ara promoters in Escherichia coli. Biochemistry and Molecular Biology Education. 2007;35(2):133–7.

4.	Jaskólska M, Adams DW, Blokesch M. Two defence systems eliminate plasmids from seventh pandemic Vibrio cholerae. Nature. 2022 Apr;604(7905):323–9.

5.	Truong TT, Vettiger A, Bernhardt TG. Cell division is antagonized by the activity of peptidoglycan endopeptidases that promote cell elongation. Molecular Microbiology. 2020;114(6):966–78.
