# Supplementary material for: Genetic interaction mapping reveals functional relationships between peptidoglycan endopeptidases and carboxypeptidases
Source: PLoS Genet. 2024 Apr 10;20(4):e1011234. doi: 10.1371/journal.pgen.1011234 (PMC11034669; doi:10.1371/journal.pgen.1011234)
Supplement: Attachment — Submitted filename: Response to Reviewer comments.docx [file pgen.1011234.s024.docx]

Reviewer #1: The authors have dealt with most of my comments in a satisfactory way. Nevertheless,
my original main criticism still stands – the authors claim a genetic interaction between DacA1 and Endopeptidases – notably ShyA. I think that the 'interaction' is based on environmental conditions. In my view, the interaction can be explained by the salt sensitivity of ShyA. This is not something that should be explored in a separate paper - especially since the authors also speculate on the role of the SMF in the observed phenotypes.
High salt is known to increase hydrophobic interactions – a disruption of such interactions, as in the L109K mutant – activates ShyA. Thus, at low salt, ShyA is more active. ShyA prefers tetra- over pentapeptide PG (earlier work by the authors). Thus, it would follow that the less active ShyA at high salt cannot sufficiently process peptidoglycan when DacA1 is absent – because of the increase in pentapeptide PG, whereas at low salt ShyA is active enough to sufficiently process PG in the absence of DacA1. The hyperactive ShyA then is only tolerated when there is more pentapeptide PG since that is cleaved less efficiently. All the other suppressor mutations also point to this – increases in pentapeptide PG are compensated for by downregulation of PG synthesis or upregulation of PG degradation.
The authors speculate on a role for the SMF – while providing some data that hints in this direction, this is far from conclusive, and in my view this should be considered together with the effects of salt on ShyA activity.

We thank reviewer 1 for their constructive criticism. As a result, we have expanded on the possible effect of salt concentration on ShyA activity in our discussion (Line 430).

Reviewer #2: The authors have addressed or provided reasonable explanation to all of my previous concerns. In my opinion the revised paper has significantly improved during the review process and in its current form would be of great interest to scientific community and thus should be recommend for publication.

Reviewer #3: The revised manuscript has addressed my main comments and most of those of the other reviewers.
With a few minor corrections listed below, I believe the manuscript can be accepted.

Minor comments:
Lines 148, 526, 546, 835: More a suggestion, but for Remazol Brilliant Blue (RBB), keep the same name/abbreviation along the manuscript, not Remazol brilliant or Remazol-Blue. Has been changed throughout to Remazol Brilliant Blue
Line 225: murAP122S instead of murA P1222 Edited
Line 280: MurC instead of fMurC Edited
Line 292: Please verify if any letters are appearing as barred in the PDF (e.g.: partially) Edited
Line 580: The sentence is incomplete: “Genomic DNA from was extracted…”. Edited
Line 603: anti- αRpoA instead of anti- αRpoI Edited
Line 633: acetylglucosamine instead of “acetylglucodamine” Edited
Legends of supplemental figures:
Lines 924, 940, 942, 955 : A) and B), etc.. in bold Edited
Line 956: remove the second B) Removed
